# Interspecific synchrony of seabird population growth rate and breeding success

**DOI:** 10.1002/ece3.592

**Published:** 2013-05-30

**Authors:** James P W Robinson, Maria Dornelas, Alfredo F Ojanguren

**Affiliations:** 1 Department of Biology, University of VictoriaVictoria, British Columbia, Canada; 2 Centre for Biological Diversity, Scottish Oceans Institute, University of St AndrewsSt Andrews, Fife, U.K; 3 CESAM, DeBio, University of AveiroPortugal

**Keywords:** Community variability, environmental forcing, extreme events, positive correlation, stability

## Abstract

Environmental variability can destabilize communities by causing correlated interspecific fluctuations that weaken the portfolio effect, yet evidence of such a mechanism is rare in natural systems. Here, we ask whether the population dynamics of similar sympatric species of a seabird breeding community are synchronized, and if these species have similar exceptional responses to environmental variation. We used a 24-year time series of the breeding success and population growth rate of a marine top predator species group to assess the degree of synchrony between species demography. We then developed a novel method to examine the species group – all species combined – response to environmental variability, in particular, whether multiple species experience similar, pronounced fluctuations in their demography. Multiple species were positively correlated in breeding success and growth rate. Evidence of “exceptional” years was found, where the species group experienced pronounced fluctuations in their demography. The synchronous response of the species group was negatively correlated with winter sea surface temperature of the preceding year for both growth rate and breeding success. We present evidence for synchronous, exceptional responses of a species group that are driven by environmental variation. Such species covariation destabilizes communities by reducing the portfolio effect, and such exceptional responses may increase the risk of a state change in this community. Our understanding of the future responses to environmental change requires an increased focus on the short-term fluctuations in demography that are driven by extreme environmental variability.

## Introduction

Natural variability in the dynamics of ecological communities can make detection of impacts of global change difficult (Magurran and Dornelas 2010), and yet with the correct tools we may use this variation to answer questions of community dynamics and predict future responses to change (Micheli et al. 1999). Species populations fluctuate through time for natural and anthropogenic causes, and the observed variation may be attributed to three mechanisms. Populations may fluctuate independently, when species respond to different abiotic drivers. Populations may correlate negatively where patterns in growth and decline in populations compensate each other (Micheli et al. 1999; Gonzalez and Loreau 2009). Environmental forcing can also synchronize populations to fluctuate in time with one another (Loreau and de Mazancourt 2008). Independent fluctuations and negative correlations dampen fluctuations at the assemblage scale, whereas synchronized fluctuations enhance them. Pronounced fluctuations in species demography may increase the chance of pushing an ecosystem past a tipping point and risk a shift to an alternative state (Scheffer et al. 2001). Extreme fluctuations can make maintaining ecosystem state, and therefore function, increasingly difficult. Here, we analyze a time series of seabird populations with the goal of identifying synchrony in extreme fluctuations.

Research into population synchrony has generally focused on spatial synchrony, whereby geographically distinct populations fluctuate in time, and both empirical and theoretical studies have identified strong evidence for synchrony at varying spatial scales (Bjornstad et al. 1999; Cazelles and Stone 2003; Liebhold et al. 2004; Grosbois et al. 2009). By instead focusing on sympatric synchrony we can reveal local responses of a community or guild. For example, theoretical studies have suggested that compensatory dynamics – where decline in one population is compensated for by increase in another – can stabilize communities (Tilman et al. 2006; Gonzalez and Loreau 2009). However, support for the prevalence of this mechanism in natural systems is weak, as illustrated by Houlahan et al.'s (2007) finding of only positive covariance across communities of various taxa, and recent work that suggests environmental disturbances can result in correlated interspecific fluctuations (Keitt 2008; Gouhier et al. 2010; Pandit et al. 2013). Studies of synchrony can contribute to our understanding of ecosystem stability by revealing the strength and direction of species interactions, as well as their responses to environmental change.

Interspecific synchrony can be driven by (1) shared climate or resources, where correlated environmental factors impact on population dynamics, and (2) shared predation, where either the functional responses or population variation in a predator synchronizes multiple prey species (Liebhold et al. 2004; Kvasnes et al. 2010). Loreau and de Mazancourt (2008) used theoretical models to find that similar species are strongly synchronized by drivers of community regulation and environmental forcing. The authors suggest that species exploiting the same resources will be similarly affected by their environment, and thus should fluctuate in synchrony.

Studying the extent of synchrony in a community can help assess the stability of an ecosystem and its vulnerability to phase shifts. The portfolio effect describes how diversity or species richness can stabilize fluctuations in community or ecosystem function (Kremen 2005) and is characterized by asynchronous fluctuations in species abundance. For example, between species competitive interactions, or variable responses to climate effects (response diversity), can result in negative correlations (a form of asynchrony) and increase the strength of the portfolio effect (Thibaut et al. 2012). Evidence of synchrony reveals a weakening of the portfolio effect, and therefore may indicate reduced ecosystem stability (Loreau 2010).

A second approach to understanding ecosystem stability via synchrony concerns extreme responses to environmental variability, and the idea that disturbance can induce interspecific synchrony. Analysis of such a phenomenon appears to be limited, although Keitt (2008) identified whole community synchrony in response to environmental disturbance, and Gouhier et al. (2010) showed that strong environmental noise synchronized consumers. Studies of ecological stability need to consider such responses to environmental change, and Keitt (2008) calls for development of methods suited to studying such exceptional synchrony. Indeed, extreme climate events may cause pronounced fluctuations in population dynamics, altering species interactions and reducing ecosystem stability (Ives and Carpenter 2007), and potentially changing ecosystem state (Scheffer et al. 2001). Future anthropogenic change will increase environmental variation and the severity of extreme events (Jentsch et al. 2007). Empirical studies of synchrony can complement theory in exploring how community stability is affected by such strong environmental variation. Here, we attempt to explore multiple species responses to climate variability by identifying synchrony in similar sympatric species.

This study investigates synchrony in the population growth rate and breeding success of a group of seabird species in a colony on the east coast of Scotland. Evidence for environment forcing is tested using the oceanographic factors of the North Atlantic Oscillation (NAO) and sea surface temperature (SST). Both climate variables have been shown to impact seabird demography through direct effects on survival and indirect effects on prey availability (Durant et al. 2005; Harris et al. 2005a; Sandvik et al. 2005). Lahoz-Monfort et al. (2011, 2013) and Wanless et al. (2009) studied the same seabird group and identified some interspecific synchrony of adult survival, breeding success and breeding phenology, respectively, but in a smaller number of species. We attempt to understand the dynamics of the seabird guild by using a new method to specifically identify years with similar pronounced fluctuations in demography, and by expanding the number of species analyzed. We term these “exceptional years” in the time series, which could potentially result in major changes at the local guild level. Our study hypothesizes that the breeding success and growth rates of all species are synchronized, and that species will respond similarly to strong environmental variation in a given year. We expect breeding success to be more synchronous than growth rate because all species share the same environment during breeding, but winter in different locations (Dunnet et al. 1990; Harris et al. 2005a). Additionally, environmental forcing is investigated by testing the hypothesis that breeding success and growth rate of each species, and the species group, are correlated with climate variables.

## Materials and Methods

Seabird breeding and count data were obtained from the Seabird Monitoring Project (SMP) for species at the Isle of May colony on the Southeast coast of Scotland. Count estimates were available from 1986 to 2009 and breeding success from 1986 to 2010. wNAO Index Data were provided by the Climate Analysis Section, NC, AR, Boulder, U.S.A. (Hurrell 1995) at http://www.cgd.ucar.edu/cas/jhurrel/ (date accessed: 06 July 2011). wNAO is the December–March mean value for year *Y*_*t*_, where December is *Y*_*t*-1_. SST data were sourced from HadSST2 (British Atmospheric Data Centre) and extracted from http://wxweb.meteostar.com/SST/ (date accessed: 30 June 2011). SST anomaly data are averaged for February–March (hereafter wSST) and taken at 55 to 60 N × 0 to 5 W, an area including the Isle of May. Both wNAO and wSST of the previous year were included in analyses (wNAO_1, wSST_1) to investigate lagged indirect effects.

Data were collected following the methods described by Walsh et al. (1995). Breeding success data were collected by the Centre for Ecology and Hydrology (CEH) by making repeated checks of nests within sample plots for each species. It is calculated as the average number of chicks fledged across the plots (Harris et al. 2005b). Count data were collected by the SMP, using full colony counts and sample plots. Each year of the count data is strongly affected by the previous year's value because many individuals survive from 1 year to the next, causing temporal autocorrelation. The time series was differenced to remove the autocorrelation (i.e., the difference in count between each year) (Bjornstad et al. 1999; Brown et al. 2011). The differenced counts for each species were then divided by the species count for the preceding year to give the per capita instantaneous population growth rate (hereafter, growth rate),


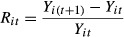


where *R*_*it*_ is the growth rate for species *i* at time *t* and *Y*_*it*_ is the count for species *i* at time *t* (Pandit et al. 2013). We analyzed growth rates to prevent very abundant species from monopolizing changes in the group as a whole, and because population growth is more strongly synchronized than population size (Loreau and de Mazancourt 2008). Atlantic puffin (*Fratercula arctica*) was omitted from these analyses because yearly population estimates were not available from SMP. Breeding success data are calculated from point estimates and thus subject to some uncertainty; the count data are subject to observation error. However, given the extensive use of the SMP dataset by CEH we feel the data are precise and accurately reflect the seabird group demography.

Synchrony between species and across time was investigated using a combination of methods. First, data were arranged in a matrix of *i* species (rows) by *j* years (columns) where *y*_*ij*_ is the value (either growth rate or breeding success) of species *i* at year *j* for all parameters. We then used three approaches: (1) analyzing correlations between all possible pairs of species, (2) summing species responses to analyze the group of species as a whole and identify exceptional fluctuations, and (3) analyzing correlations between environmental variables with each species individually and with the group response. These are alternative methods to studying multispecies demography than those employed in past studies of Isle of May seabirds (Wanless et al. 2009; Lahoz-Monfort et al. 2011, 2013).

For the first approach, Pearson correlations between species for growth rate and for breeding success were calculated to identify species with similar or inverse temporal patterns, therefore assessing the degree of synchrony between species. The method is commonly used in studies of synchrony (e.g., Bjornstad et al. 1999; Kvasnes et al. 2010), though was not employed in previous work on synchrony in the Isle of May seabirds (e.g., Lahoz-Monfort et al. 2011, 2013). The null model tested was that each correlation between species was independent between species and through time. To avoid the problem of nonindependence between time series the analyses were bootstrapped (Efron and Tibshirani 1993; Bjornstad et al. 1999; Liebhold et al. 2004; Lillegård et al. 2005), where the parameter (either growth rate or breeding success) for each species was resampled for each year without replacement and a Pearson correlation matrix generated for each of 1000 permutations. The null model was rejected when the observed value was outside the confidence interval obtained from sorting the permutations. We present results for a significance level of 0.05 (95% confidence intervals) and also corrected for multiple comparisons using the sequential Bonferroni method (Rice 1988).

For the second approach, each demographic parameter was summed for all species by year to identify exceptional years for the species group, when the species group demographic rates were more synchronous than expected by chance. The null model tested was that growth rate and breeding success were independent among species and through time. Again, the values for each species were resampled without replacement and each year summed for all species, for a total of 1000 permutations. The null model was rejected when the observed value was outside the 95% confidence interval obtained from sorting the permutations (*P* < 0.025).

For the relationship between population variables and environmental factors, a Pearson correlation matrix was used to identify single-species and species group relationships with climate variables. The significance level was set at 0.05 (95% confidence levels) and correction was determined using the sequential Bonferroni method (Rice 1988).

## Results

**Figure 1:**
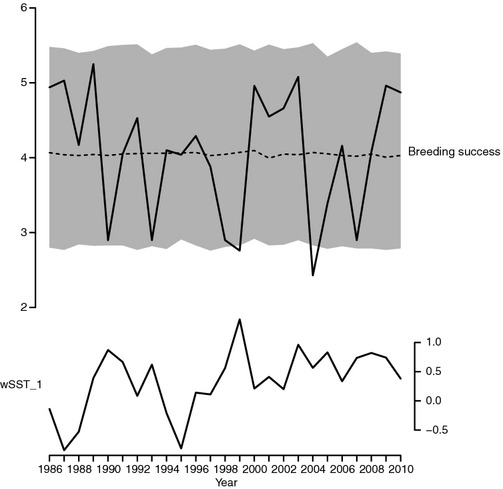
Top graph is the species group growth rate. 95% confidence intervals are shaded gray. The dotted line is the median and the solid line is the observed data. Bottom graph is lagged SST (SST_1)

**Figure 2:**
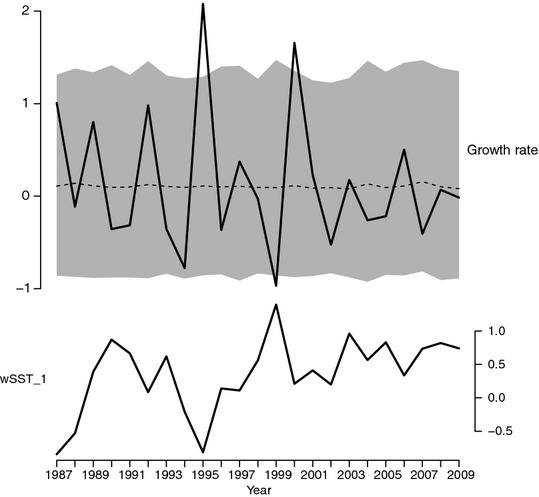
Top graph is the species group breeding success. 95% confidence intervals are shaded gray. The dotted line is the median and the solid line is the observed data. Bottom graph is lagged SST (SST_1)

The Pearson correlation matrices revealed that both growth rate and breeding success were significantly synchronous between some pairs of species (Table 1). For breeding success, 7 of 15 possible correlations were significantly positive, although European shag *Phalacrocorax aristotelis* was not correlated with any species. Of 10 possible correlations for growth rate, two were found to be significantly positive. There were no significant negative correlations. Synchrony was higher in breeding success than growth rate. Exceptional years for all species were identified in growth rate and breeding success. The analysis of growth rate for all species revealed 1995 (*P* = 0.004) and 2000 (*P* = 0.006) to be positive years, and 1999 (*P* = 0.018) to be a negative year for the species group growth rate (Fig. 1). Analyzing all species breeding success revealed 1999 (*P* = 0.021) and 2004 (*P* = 0.003) to be significantly negative years for the species group (Fig. 2).

**Table 1:** Matrix of Pearson correlation coefficients between species for growth rates (below diagonal) and breeding success (above diagonal)

Common name	Common guillemot (CG)	Fulmar (F)	Black-legged kittiwake (K)	Razorbill (R)	European shag (S)	Atlantic puffin (AP)

Latin name	*Uria aalge*	*Fulmaris glacialis*	*Rissa tridactyla*	*Alca torda*	*Phalacrocorax aristotelis*	*Fratercula arctica*
CG		**0.53**	0.28	**0.52**	−0.16	0.66*
F	0.34		**0.41**	0.32	0.04	0.64*
K	0.33	0.08		0.22	0.11	0.60*
R	**0.44**	0.24	0.44		−0.07	**0.49**
S	**0.46**	0.27	0.09	−0.04		0.01
AP	–	–	–	**–**	–	

Bold values indicate significant values for the permutation method. Significant values by sequential Bonferroni are indicated with a star.

A negative effect of lagged wSST was found for growth rate of kittiwake, and wNAO was positively correlated with razorbill *Alca torda* growth rate (Table 2). Breeding success of common guillemot *Uria aalge*, kittiwake *Rissa tridactyla*, razorbill, and Atlantic puffin *Fratercula arctica* was negatively correlated with 1 year lagged wSST (Table 2). Atlantic puffin was also negatively correlated with wSST of the same year (no lag). The summed species analysis identified a negative effect of lagged wSST on growth rate and breeding success (Table 2). No other climate variables had significant relationships with the summed species parameters. The species group growth rate and breeding success are presented with lagged SST in Figures 1, 2.

**Table 2:** Pearson correlations for growth rate and breeding success with climate variables for all species

Species		Growth rate				Breeding success			
		
Common name	Latin name	wSST	wSST_1	wNAO	wNAO_1	wSST	wSST_1	wNAO	wNAO_1
Common guillemot	*Uria aalge*	−0.17	−0.41	0.31	0.22	−0.36	−**0.40**	0.09	0.24
Fulmar	*Fulmaris glacialis*	−0.22	−0.41	0.01	−0.06	−0.36	−0.32	0.12	−0.04
Black-legged kittiwake	*Rissa tridactyla*	−0.06	−**0.50**	0.31	−0.04	−0.34	−**0.42**	−0.03	−0.22
Razorbill	*Alca torda*	0.02	−0.29	**0.44**	−0.10	−0.22	−**0.40**	0.28	0.09
European shag	*Phalacrocorax aristotelis*	0.19	−0.25	−0.11	0.03	0.16	0.01	−0.35	−0.07
Atlantic puffin	*Fratercula arctica*	–	–	–	–	−0.49*	−0.64*	−0.07	0.15
All species		0.02	−0.55*	0.23	0.00	−0.24	−**0.41**	−0.20	−0.07

Bold values indicate significant values for the permutation method. Significant values by sequential Bonferroni are indicated with a star.

## Discussion

Correlation analysis identified positive correlations in breeding success between five species (seven pairs), and less synchrony between growth rates. Additionally, the absence of negative correlations is consistent with previous studies (e.g., Houlahan et al. 2007; Thibaut et al. 2012) and justifies the focus on synchrony rather than compensatory dynamics among the species. More extensive synchrony in breeding success than growth rate can be explained by the species sharing diets and breeding grounds, but some wintering in different locations (Dunnet et al. 1990; Harris et al. 2005a; Fauchald et al. 2011). While the species share prey species, the lack of complete synchrony in the group breeding success (not all species positively correlated) is likely due to differences in sensitivity to prey availability (Furness and Tasker 2000) and differences in feeding strategy (i.e., diving depth) (Camphuysen and Webb 1999). The low correlations in the breeding success of shag *Phalacrocorax aristotelis* were potentially due to their high clutch size and occasional repeat breeding: both of which are not life-history traits in the other study species (Frederiksen et al. 2008). In addition, an alternative approach that explicitly modeled interspecific variation in clutch size was successful in identifying multispecies synchrony between the same species (Lahoz-Monfort et al. 2013). We suggest that the group of species is similarly affected by local environmental change, a finding supported by our second approach.

Analysis of the exceptional years identified another form of synchrony, where breeding success and growth rate of the species group were both found to have years of exceptional performances (both positive and negative responses). Despite the limited pairwise synchrony in growth rates identified by the Pearson correlation matrix, the summed species approach revealed three independent years of exceptional growth rates for all species. Analyzing the species group allows changes at the guild level to be observed, and can offer a measure of changes in the community that may be masked in single-species studies, or masked by more traditional measures of correlation between species. Additionally, the method is particularly important in developing our understanding of community dynamics and stability. Ives and Carpenter (2007) suggest that stability might be measured by the amplitude of fluctuations of some system property: our study offers another method of examining system stability, though it is likely that top predator demography does not accurately reflect community stability in the North Sea. The approach of identifying exceptional years focuses on separating background variability from variability with consequences for the local guild, and complements study of long-term trends and of single-species dynamics.

Single-species tests of correlations between climate and demography revealed some significant relationships between species demography and climate variables, though no single parameter was correlated for every species. Evidence for a negative relationship between seabird breeding performance and lagged SST has previously been reported (Durant et al. 2003, 2005; Frederiksen et al. 2007). The positive correlation between NAO and razorbill *Alca torda* growth rate is novel, though was not shown for other species. However, by analyzing the summed species group we investigate trends at the guild level and can reveal drivers of multiple species. We find a negative correlation between lagged winter SST and the species group demography, suggesting a degree of environmental forcing on the seabird community. Delayed maturity of juveniles may cause synchrony in growth rates. Juveniles can delay first breeding and thus their return to the colony in years of poor resources, as shown for fulmar by Thompson and Ollason (2001). For breeding success, previous studies have found seabird performance to strongly correlate with age 1 sandeel availability (Durant et al. 2005), driven by a negative correlation between SST and sandeel recruitment (Arnott and Ruxton 2002). Despite variation in the sensitivity of seabird species to changing prey abundance (Furness and Tasker 2000), it is likely that SST drives variation in the breeding success of multiple species via its influence on seabird prey.

Identifying environmental forcing and occurrence of exceptional synchronized fluctuations suggests that species may have synchronous responses to extreme disturbances. The portfolio effect protects communities against change (Cottingham et al. 2001) and is strengthened by asynchrony (Thibaut et al. 2012). We expect that increased synchrony during exceptional years will make ecosystems vulnerable to state changes. This highlights the importance of extreme disturbances. Future increases in the frequency and strength of extreme events may therefore increase the likelihood of a phase shift in a community strongly influenced by environmental forcing.

By studying synchrony we can better understand community interactions and ecosystem responses to climate change. Competitive theory predicts ecologically similar species should display compensatory dynamics (Ranta et al. 2008), yet we find evidence for synchrony in a seabird group, and suggest that environmental drivers force covariation. While we acknowledge that the study species do differ in their life-history strategies, particularly in feeding mode, we show that by examining the group response we reveal a shared response to prey availability that was not observed at the species level. Further work might employ methods that incorporate interspecific variation in life-history traits, such as Lahoz-Monfort et al.'s (2013) approach to modeling seabird productivity, while exploring the concept of exceptional years. Additionally, we find further evidence that environmental perturbations can induce whole community synchrony (Keitt 2008; Gouhier et al. 2010), and suggest that species covariation should be further explored in the context of community stability and the portfolio effect. Studies of environmental change effects should consider the magnitude of fluctuations as well as examining trends and long-term projections. Identifying exceptional years improves our ability to identify potential times of drastic change, and highlights potential risks to the stability of ecosystem function. For example, exceptional years may significantly boost population abundance of a local guild, but ecosystem function may be significantly degraded by years of poor performance. By examining the demographic fluctuations of multiple species we learn about community and ecosystem dynamics, and deepen our understanding of the impact of global change.

## References

[b1] Arnott SA, Ruxton GD (2002). Sandeel recruitment in the North Sea: demographic, climatic and trophic effects. Mar. Ecol. Prog. Ser.

[b2] Bjornstad ON, Ims RA, Lambin X (1999). Spatial population dynamics: analyzing patterns and processes of population synchrony. Trends Ecol. Evol.

[b3] Brown CJ, Schoeman DS, Sydeman WJ, Brander K, Buckley LB, Burrows M (2011). Quantitative approaches in climate change ecology. Glob. Change Biol.

[b4] Camphuysen K, Webb A (1999). Multi-species feeding associations in North Sea seabirds: jointly exploiting a patchy environment. Ardea.

[b5] Cazelles B, Stone L (2003). Detection of imperfect population synchrony in an uncertain world. J. Anim. Ecol.

[b6] Cottingham KL, Brown BL, Lennon JT (2001). Biodiversity may regulate the temporal variability of ecological systems. Ecol. Lett.

[b7] Dunnet GM, Furness RW, Tasker ML, Becker PH (1990). Seabird ecology in the North Sea. Neth. J. Sea Res.

[b8] Durant JM, Anker-Nilssen T, Stenseth NChr (2003). Trophic interactions under climate fluctuations: the Atlantic puffin as an example. Proc. Biol. Sci.

[b9] Durant JM, Stenseth NChr, Anker-Nilssen T, Harris MP, Thompson PM, Wanless S, Stenseth NChr (2005). Marine birds and climate fluctuation in the North Atlantic. Marine ecosystems and climate variation: the North Atlantic: a comparative perspective.

[b10] Efron B, Tibshirani R (1993). An introduction to the bootstrap.

[b11] Fauchald P, Skov H, Skern-Mauritzen M, Hausner VH, Johns D, Tveraa T (2011). Scale-dependent response diversity of seabirds to prey in the North Sea. Ecology.

[b12] Frederiksen M, Edwards M, Mavor RA, Wanless S (2007). Regional and annual variation in black-legged kittiwake breeding productivity is related to sea surface temperature. Mar. Ecol. Prog. Ser.

[b13] Frederiksen M, Daunt F, Harris MP, Wanless S (2008). The demographic impact of extreme events: stochastic weather drives survival and population dynamics in a long-lived seabird. J. Anim. Ecol.

[b14] Furness RW, Tasker ML (2000). Seabird-fishery interactions: quantifying the sensitivity of seabirds to reductions in sandeel abundance, and identification of key areas for sensitive seabirds in the North Sea. Mar. Ecol. Prog. Ser.

[b15] Gonzalez A, Loreau M (2009). The causes and consequences of compensatory dynamics in ecological communities. Annu. Rev. Ecol. Evol. Syst.

[b16] Gouhier TC, Guichard F, Gonzalez A (2010). Synchrony and stability of food webs in metacommunities. Am. Nat.

[b17] Grosbois V, Harris MP, Anker-Nilssen T, McCleery RH, Shaw DN, Morgan BJT (2009). Modeling survival at multi-population scales using mark–recapture data. Ecology.

[b18] Harris MP, Anker-Nilssen T, McCleery RH, Erikstad KE, Shaw DN, Grosbois V (2005a). Effect of wintering area and climate on the survival of adult Atlantic puffins Fratercula arctica in the eastern Atlantic. Mar. Ecol. Prog. Ser.

[b19] Harris MP, Wanless S, Murray S, Mackley E (2005b). Isle of May seabird studies in 2004. JNCC Report.

[b20] Houlahan JE, Currie DJ, Cumming GS, Ernest SKM, Findlay CS, Fuhlendorf SD (2007). Compensatory dynamics are rare in natural ecological communities. Proc. Natl Acad. Sci. USA.

[b21] Hurrell JW (1995). NAO index data – climate analysis section.

[b22] Ives AR, Carpenter SR (2007). Stability and diversity of ecosystems. Science.

[b23] Jentsch A, Kreyling J, Beierkuhnlein C (2007). A new generation of climate-change experiments: events, not trends. Front. Ecol. Environ.

[b24] Keitt TH (2008). Coherent ecological dynamics induced by large-scale disturbance. Nature.

[b25] Kremen C (2005). Managing ecosystem services: what do we need to know about their ecology?. Ecol. Lett.

[b26] Kvasnes MAJ, Storaas T, Pedersen HC, Bjork S, Nilsen EB (2010). Spatial dynamics of Norwegian tetraonid populations. Ecol. Res.

[b27] Lahoz-Monfort JJ, Morgan BJT, Harris MP, Wanless S, Freeman SN (2011). A capture-recapture model for exploring multi-species synchrony in survival. Methods Ecol. Evol.

[b28] Lahoz-Monfort JJ, Morgan BJT, Harris MP, Daunt F, Wanless S, Freeman SN (2013). Breeding together: modeling synchrony in productivity in a seabird community. Ecology.

[b29] Liebhold AM, Koenig WD, Bjornstad ON (2004). Spatial synchrony in population dynamics. Annu. Rev. Ecol. Evol. Syst.

[b30] Lillegård M, Engen S, Sæther BE (2005). Bootstrap methods for estimating spatial synchrony of fluctuating populations. Oikos.

[b31] Loreau M (2010). From populations to ecosystems: theoretical foundations for a new ecological synthesis.

[b32] Loreau M, de Mazancourt C (2008). Species synchrony and its drivers: neutral and nonneutral community dynamics in fluctuating environments. Am. Nat.

[b33] Magurran AE, Dornelas M (2010). Biological diversity in a changing world. Philos. Trans. R. Soc. B Biol. Sci.

[b34] Micheli F, Cottingham KL, Bascompte J, Bjornstad ON, Eckert GL, Fischer JM (1999). The dual nature of community variability. Oikos.

[b35] Pandit SN, Kolasa J, Cottenie K (2013). Population synchrony decreases with richness and increases with environmental fluctuations in an experimental metacommunity. Oecologia.

[b36] Ranta E, Kaitala V, Fowler MS, Laakso J, Ruokolainen L, O'Hara R (2008). Detecting compensatory dynamics in competitive communities under environmental forcing. Oikos.

[b37] Rice W (1988). Analysing tables of statistical tests. Evolution.

[b38] Sandvik H, Erikstad KE, Barrett RT, Yoccoz NG (2005). The effect of climate on adult survival in five species of North Atlantic seabirds. J. Anim. Ecol.

[b39] Scheffer M, Carpenter S, Foley JA, Folke C, Walker B (2001). Catastrophic shifts in ecosystems. Nature.

[b40] Thibaut LM, Connolly SR, Sweatman HPA (2012). Diversity and stability of herbivorous fishes on coral reefs. Ecology.

[b41] Thompson PM, Ollason JC (2001). Lagged effects of ocean climate change on fulmar population dynamics. Nature.

[b42] Tilman D, Reich PB, Knops JMH (2006). Biodiversity and ecosystem stability in a decade-long grassland experiment. Nature.

[b43] Walsh PM, Halley DJ, Harris MP, Sim A, del Nevo IMW, Tasker ML (1995). Seabird monitoring handbook for Britain and Ireland.

[b44] Wanless S, Frederiksen M, Walton J, Harris MP (2009). Long-term changes in breeding phenology at two seabird colonies in the western North Sea. Ibis.

